# Supplementary material for: Prepared for the expected but unready for the unexpected: Unmet distractor expectations slow braking responsiveness but improve lane-keeping precision in a virtual driving simulation
Source: PLoS One. 2025 Dec 26;20(12):e0338124. doi: 10.1371/journal.pone.0338124 (PMC12742790; doi:10.1371/journal.pone.0338124)
Supplement: S2 File — (PDF) [file pone.0338124.s002.pdf]

Prepared for the expected but unready for the unexpected: unmet distractor expectations slow braking responsiveness but improve lane-keeping precision in a virtual driving simulation

SUPPLEMENTARY MATERIALS

It might be argued that variations in vigilance and/or cognitive demand across blocks influenced Braking RTs, as the Mixed-Conjunction block, being more cognitively demanding, might have sustained attention (or general attentional engagement) more heavily. Although we cannot entirely rule out this possibility due to the absence of a direct measure of sustained attention in our setup, we argue that it cannot fully account for the slower RTs. Increased vigilance typically speeds RTs, particularly at the beginning of a block. If the modulation of RTs were entirely attributable to fluctuations in vigilance, we would expect them to progressively slow as the block unfolds, an effect referred to as vigilance decrement [1]. Furthermore, if vigilance levels vary with cognitive demand, the rate of vigilance decrement would be expected to differ across Pure, Feature, and Conjunction Search blocks. Differences between TP-DA and TP-DP trials may also emerge, as their physical characteristics are likely to elicit distinct responses. To test this prediction, we conducted two independent mixed-effects linear regression models, one for TP-DA trials and one for TP-DP trials.

TP-DA trials

First, we ran three independent mixed-effects linear regression models, each including the interaction between Block type and, respectively, the linear, quadratic, and cubic polynomial contrasts for the Trial sequence variable as fixed effects, with the by-subject intercept included as a random component.

Model comparison (see Table S1) indicates that the quadratic model is the most parsimonious. However, although adding the cubic term does not significantly improve the model (though it approaches significance), the cubic model provides a better fit to the data, balancing model fit and complexity, according to the Akaike Information Criterion (AIC). We thus selected the latter model for subsequent analyses.

Model	k	AIC	χ^2	Df	*p*
Linear	8	-1438.4			
Quadratic	11	-1444.3	11.8767	3	.008
Cubic	14	-1445.2	6.9194	3	.074

Table S1. Model comparison.

Results indicate a significant main effect of Block Type ($F_{(1, 2073.0)} = 78.9$, $p < .001$), as well as for the cubic polynomial for Trial sequence ($F_{(3, 2073.1)} = 4.27$, $p = .005$), whereas their interaction does not reach statistical significance ($F_{(6, 2073.0)} = 1.56$, $p = .155$). Specifically, the analysis of the orthogonal polynomial contrasts revealed a significant cubic component ($\beta = -0.58$, SE = 0.29, $t_{(2073)} = -2.00$, $p = .045$), net of Block Type. In contrast, both the linear and quadratic components did not reach significance ($p > .05$). Visual inspection of the graph (Fig. S1) revealed that RTs decrease progressively across trials in both Mixed-Conjuntion and Mixed-Feature Blocks. In contrast, in the Pure Block, they initially decrease but eventually returned to the starting level by the end of the block. This pattern suggests that, in the Mixed Blocks, participants may have begun to anticipate TP-DA trials as the block unfolded, thereby reducing the negative impact of unmet distractor-related expectations.

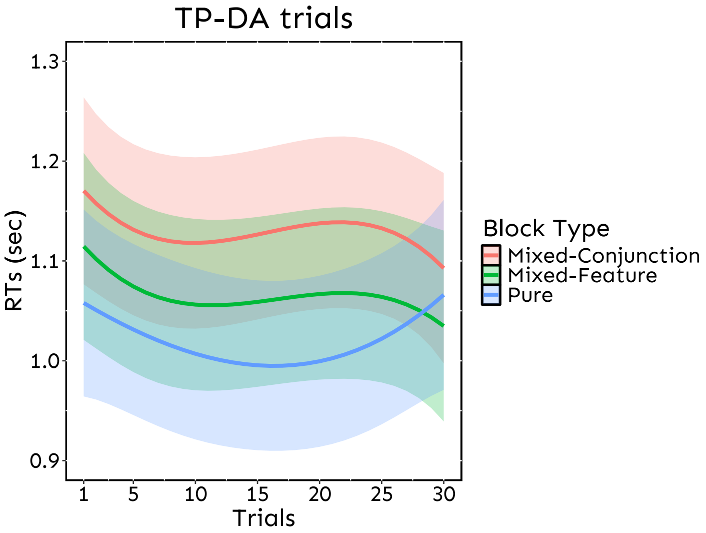

Fig. S1. RTs as a function of Block and Trial sequence

To more precisely and quantitatively characterize these results, we ran three independent mixed-effects linear regression models (one for each block). Each model included the interaction between Block type and polynomial contrasts of the Trial sequence variable (up to the cubic term) as fixed effects, with a random intercept for subjects. The analysis of the orthogonal polynomial contrasts revealed a significant cubic component for both the Mixed-Conjunction ($\beta = -0.33$, SE = 0.17, $t_{(669)} = -1.98$, $p = .048$) and the Mixed-Feature ($\beta = -0.28$, SE = 0.14, $t_{(677)} = -2.03$, $p = .043$), and a significant quadratic trend for the Pure Block ($\beta = 0.54$, SE = 0.15, $t_{(681)} = 3.71$, $p < .001$).

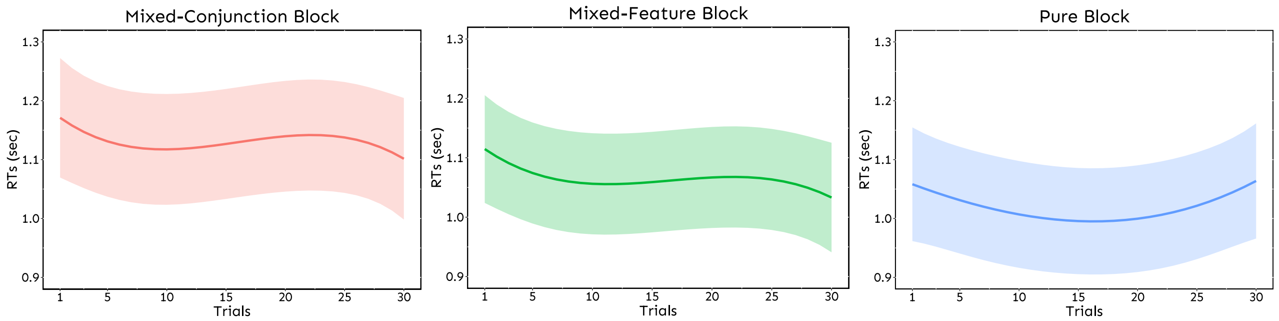

Fig. S2. RTs as a function of Trial sequence for each Block

RTs decrease progressively across trials in both Mixed-Conjuntion and Mixed-Feature Blocks. In contrast, in the Pure Block, they initially decrease but eventually returned to the starting level by the end of the block. This pattern suggests that, in the Mixed Blocks, participants may have begun to anticipate TP-DA trials as the block unfolded, thereby reducing the negative impact of unmet distractor-related expectations.

This analysis confirms a progressive and nonlinear decrease in RTs across trials in both the Mixed-Conjunction and Mixed-Feature blocks, consistent with the hypothesis of proactive anticipation of TP-DA trials by the end of the blocks. In contrast, the "U-shaped" pattern observed in the Pure block, which included only DA trials, may be explained by reduced vigilance toward the end of the block, induced by its monotonic nature.

It might be argued that RTs already differed between blocks at trial 1. However, it should be noted that each block was preceded by a one-minute training period, allowing participants to preview the task and develop a distractor expectation set. This familiarization phase likely contributed to establishing a baseline before the actual experimental session began.

TP-DP trials

First, we ran three independent mixed-effects linear regression models, each including the interaction between Block type and, respectively, the linear, quadratic, and cubic polynomial contrasts for the Trial sequence variable as fixed effects, with the by-subject intercept included as a random component.

Model comparison (see Table S2) indicates that the quadratic model is the most parsimonious and it provides a better fit to the data, balancing model fit and complexity, according to the Akaike Information Criterion (AIC). We thus selected this model for subsequent analyses.

Model	k	AIC	χ^2	Df	p
Linear	6	-2090.5			
Quadratic	8	-2094.1	7.5408	2	0.023
Cubic	10	-2091.2	1.1740	2	0.556

Table S2. Model comparison.

Results indicate a significant main effect of Block Type ($F_{(1, 2778.0)} = 167.29$, $p < .001$), and a significant interaction between Block Type the quadratic trend for Trial sequence ($F_{(3, 2778.0)} = 3.65$, $p = .026$). Specifically, the analysis of the orthogonal polynomial contrasts revealed that the Mixed-Feature Block entails a significant quadratic component as compared to the Mixed-Conjunction one ($\beta = 0.65$, SE = 0.32, $t_{(2778)} = 2.00$, $p = .046$).

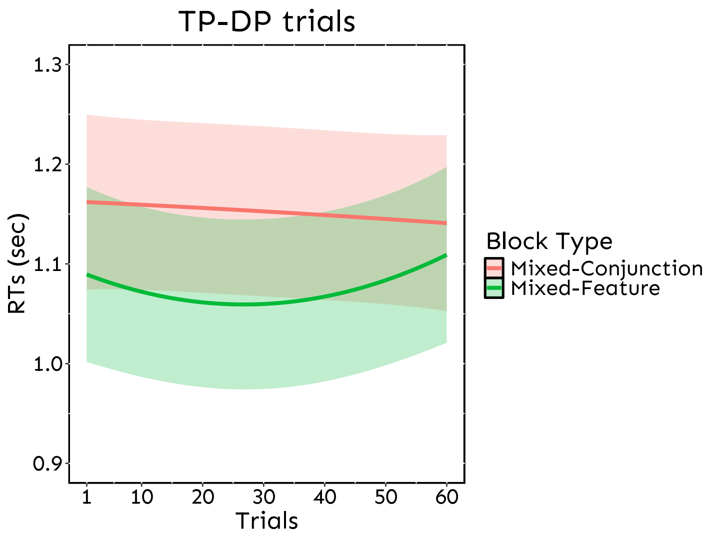

Fig. S3. RTs as a function of Block and Trial sequence

To more precisely and quantitatively characterize these results, we ran two independent mixed-effects linear regression models (one for each block). Each model included the interaction between Block type and polynomial contrasts of the Trial sequence variable (up to the quadratic term) as fixed effects, with a random intercept for subjects. The analysis of the orthogonal polynomial contrasts revealed a significant quadratic component for both the Mixed-Feature (β = 0.41, SE = 0.15, $t_{(1382)}$ = 2.75, p = .006) and non-significant trends for the Mixed-Conjunction one (p > .05).

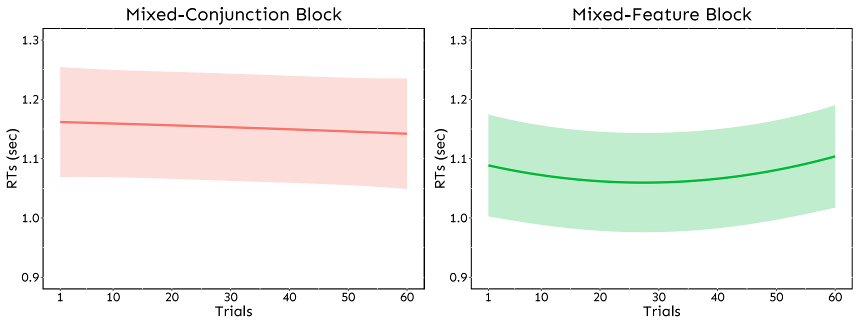

Fig. S4. RTs as a function of Trial sequence for each Block

While the difference between the two blocks may be attributable to differences in cognitive demands, we hypothesize that this result is primarily due to our task design. Specifically, we employed an overt visual search with long stimulus exposure, which is optimal for observing variability in response times. However, although participants were instructed to respond to target stimuli as quickly as possible, the long exposure time may have allowed them to perform the task reactively (rather than proactively) suppressing distractors, which could explain the absence of learned suppression.

References

1. McCarley JS, Yamani Y. Psychometric Curves Reveal Three Mechanisms of Vigilance Decrement. Psychol Sci. 2021;32: 1675–1683. doi:10.1177/09567976211007559
