# Supplementary material for: Prepared for the expected but unready for the unexpected: Unmet distractor expectations slow braking responsiveness but improve lane-keeping precision in a virtual driving simulation
Source: PLoS One. 2025 Dec 26;20(12):e0338124. doi: 10.1371/journal.pone.0338124 (PMC12742790; doi:10.1371/journal.pone.0338124)
Supplement: S3 File — (PDF) [file pone.0338124.s003.pdf]

Prepared for the expected but unready for the unexpected: unmet distractor expectations slow braking responsiveness but improve lane-keeping precision in a virtual driving simulation

GLOSSARY

Cognitive control: The ability to effectively integrate and accommodate one's own thoughts and actions with the final aim of smoothly achieving a behavioural goal.

Conjunction search: A visual search wherein the target object can be distinguished from other objects only by combining two or more defining features.

Distraction: A mechanism unfolding when attention is dragged away from the main task by other irrelevant objects or actions.

Distractor Context Manipulation (DCM): An experimental paradigm allowing to quantify the cost associated with distractor expectation. It typically includes a Pure Block, with no distractor expectation, and a Mixed Block, with distractor expectation. By contrasting performance in physically identical trials devoid of distractors across blocks, it is possible to quantify any behavioural cost entailed by their expectation.

Feature search: A visual search wherein the target object can be distinguished from other objects by a unique defining feature.

Proactive control: A mechanism of behavioural preparation which is implemented before the occurrence of an expected distracting or interfering event.

Reactive control: A mechanism of behavioural correction which is implemented after a distracting or interfering event has occurred.

Selective attention: The cognitive process of selecting only a limited amount of all the sensory information in the environment for detailed processing.

Visual search: A paradigm wherein participants are asked to detect and respond to a relevant object (the target) among irrelevant objects (the distractors) or other non-target objects.
