# Supplementary material for: Prepared for the expected but unready for the unexpected: Unmet distractor expectations slow braking responsiveness but improve lane-keeping precision in a virtual driving simulation
Source: PLoS One. 2025 Dec 26;20(12):e0338124. doi: 10.1371/journal.pone.0338124 (PMC12742790; doi:10.1371/journal.pone.0338124)
Supplement: Attachment — Submitted filename: ResponsesToReviewers_PLOS.docx [file pone.0338124.s005.docx]

**Editor comment**:

I was able to secure two reviewers, both of whom have now provided their evaluations and comments. One conceptual issue that particularly strikes me is the following: while the manuscript frames the study as examining driving performance or aspects of driving, it appears to me that the present task does not genuinely involve driving performance in any substantive sense. Rather, it seems to be a standard cognitive experiment where driving serves merely as a superficial cover story without any genuine relation to the behavioural and cognitive demands of actual driving. I do not refer here to issues of ecological validity or to the need for more realistic settings; rather, I question whether the task meaningfully engages with the construct of driving at all. I would encourage you to reflect on this issue carefully in your revision, particularly with regard to how you frame the purpose and implications of your study. I invite you to prepare a revision of your manuscript, accompanied by a response letter in which each comment is addressed individually in a point-by-point manner.

We thank the Editor for raising this important point. We understand the concern regarding whether our task meaningfully engages driving performance. We also acknowledge that our task may limit the strength and generalizability of claims to real-world driving.

However, we also believe that, to some extent, our setup captures and approximates key and specific aspects of driving-related demands. The previous version of the manuscript may not have sufficiently conveyed such points, which prove our study’s relevance to driving-related behavioural and cognitive processes.

At the same time, we recognize that certain elements must be appropriately toned down in light of the inherent limitations of the controlled experimental settings. Specifically, using a driving simulator entails constraints such as simplified motor demands and visual stimulation, as well as a relatively high degree of experimental control, which may prevent or limit direct generalizability of the findings to real-world driving contexts.

Nonetheless, we believe that several aspects of our experimental design meaningfully aligns with real-world driving behaviour, and that the task is not simply an attentional experiment over imposed onto a driving context.

While we acknowledge that the sudden onset of road signs, as attentional stimuli, is not fully embedded within the road environment, thereby limiting their realism for the sake of experimental control, the core of our task remains primarily focused on an ecologically valid, driving-like context and the associated behavioural responses.

Compared to standard laboratory tasks (e.g., Petilli et al., 2020), which typically employed simplified and abstract visual displays, our experimental setup utilizes a markedly different setting, that is a driving simulation involving a dynamic and context-rich visual environment. Even more importantly, the response measures are inherently naturalistic driving actions that differ significantly from simple button-press responses commonly used in standard lab-based experiments.

The first critical measure, that is rapid foot braking in reaction to target objects, reflects indeed a natural, safety-relevant action.

The second critical measure, that is lane-keeping, is a response typically absent in laboratory paradigms, and it reflects our task’s goal to capture genuine driving behaviour rather than relying on experimental approximation. Importantly, this response was recorded and analyzed in the absence of any attentional stimuli unrelated to driving. This provides additional evidence that our task fully engages participants in a genuine driving-related activity, with their responses directly reflecting behavioural and cognitive demands inherent to real world-driving.

While the described approach does not aim to fully replicate all aspects of real-world driving, and involves specific constraints typical of controlled simulated environments (e.g., Getzmann et al., 2018; Karthaus et al., 2018a; Karthaus et al., 2018b; Chandrakumar et al., 2021; Facchin et al., 2023; Garcia et al., 2017; Alyan et al., 2017), the level of experimental control represents a good compromise to isolate the cognitive mechanisms underlying distractor expectation and their impact on driving behaviour within relatively ecologically valid, experimentally controlled and feasible conditions.

In the revised manuscript, we have directly addressed these issues, clarifying how our task meaningfully engages with core driving behaviors.

For example:

LINES 258-262: *“**Notably, although the described approach does not aim to reproduce all aspects of real-world driving and involves certain constraints typical of controlled simulated environments, it nevertheless allows for the investigation of cognitive processes relevant to driving under relatively ecologically valid, experimentally controlled and feasible conditions. This is an ideal compromise similarly adopted in numerous other studies investigating comparable cognitive processes [4,6,7,9,52–54].”*

LINES 596-614: *“**Despite its contributions, our study still presents a relevant limitation that must be considered. In particular, while this study represents an important step toward scenarios that more closely approximate the perceptual and motor demands of real-world driving, it still involves a relatively high level of experimental control and artificial driving demands, which constrain the direct generalizability of our findings to real-world driving contexts. It may thus be argued that our task does not reflect genuine driving performance, and that the driving component merely serves as a context for a standard lab-based experiment on the cognitive processes underlying distractor’s expectations. However, our setup utilizes a markedly different approach from standard laboratory tasks [43], that is a driving simulation featuring a dynamic and context-rich visual environment. Additionally, the response measures are inherently naturalistic driving actions, whose motor demand differs significantly from button-press responses common in laboratory settings. Specifically, the first critical measure, rapid foot braking, reflects a natural, safety-relevant action, whereas the second, lane-keeping, is typically absent in standard experimental design. Even more importantly, lane-keeping was recorded and analyzed in the absence of any attentional and visual demand unrelated to driving. Ultimately, the use of driving simulations and motor responses that reflect real-world driving task, has been recognized as a scientific standard, as evidenced by numerous studies investigating attentional processes at the wheel [4,6,9,52–54,73]. Collectively, these methodological aspects demonstrate that participants were fully engaged in a genuine driving-related task, with behavioral responses directly reflecting cognitive and motor demands inherent to real world-driving.”*

Additionally, we have substantially revised the Discussion section, where the implications for driving have been reframed more as interesting research questions to be addressed with dedicated experimental designs rather than as firm and definitive conclusions.

**Review Comments to the Author:**

**Reviewer #1:**

This manuscript explores the effects of proactive attentional control on two aspects of driving behavior—braking and lane-keeping—within a simulated environment. By adapting the Distractor Context Manipulation (DCM) paradigm to a driving context, the authors show that distractor expectations can slow down braking responses while improving trajectory stability. While this study is innovative and features an original design, the interpretation regarding the role of proactive control remains limited, as this mechanism is not directly manipulated by the authors and thus remains speculative. A substantial revision of the introduction is recommended prior to acceptance.

1. The introduction is generally well written and well referenced. The authors appropriately cite the work of Nick Gaspelin and J Geng. However, the introduction would benefit from citing recent work on negative templates in visual search (i.e., representations of distractors in working memory), particularly the contributions of Nancy Carlisle (Negative and Positive Templates: Two Forms of Cued Attentional Control, 2023) and its relevance to the recruitment of proactive control for distractor suppression (Chidharom & Carlisle, 2023, Journal of Cognitive Neuroscience).

We thank the Reviewer for the valuable suggestion. Negative templates may indeed play a key role in our study. In our paradigm, distractor features were known in advance before each block, potentially allowing participants to form a working memory representation to guide attention away from objects with features matching those of distractors. Although we did not directly manipulate these mechanisms (e.g., by contrasting negative, positive and neutral cues), and thus cannot draw firm conclusions on this point, we have acknowledged their potential role both in the Introduction:

LINES 100-105: “*When distractor features are known in advance, such processes may benefit from negative attentional templates – a representation of the distractor held in working memory – which improve search efficiency by guiding attention away from distractor features [37] (see also [38] for complementary evidence on how distractor expectations bias target templates). Neural signatures of such negative templates have revealed a robust engagement of proactive control processes prior to search onset [39].”*

and the Discussion sessions:

LINES 531-540: *“**In visual search, attentional control can be facilitated by negative attentional templates, which rely on memory representation of distractors to enhance search efficiency by guiding attention away from objects with distractor-matching features [37,39]. Moreover, search efficiency has been linked to evidence accumulation (EA) processes, in which perceptual information is progressively gathered until one of two mutually exclusive decision thresholds is reached, thereby supporting decision-making and enabling the execution of context-appropriate actions (e.g., Go/Brake - vs. No Go/No Brake) [66–68]. We hypothesized that the frequent occurrence of distractors in Mixed Blocks may have triggered the activation of task-sets related to distractor expectations – possibly as negative attentional templates – manifesting as a general enhancement in attentional processes sustained during driving.”*

2. Do vigilance levels differ across blocks in terms of accuracy and response speed? One might imagine that the Mixed-Conjunction block, being more cognitively demanding, taxes sustained attention more heavily, potentially explaining slower RTs.

A detailed response to Comments 2, 3, 4 and 6 has been provided together in response to Comment 6.

3. The cognitive demand—and thus mental effort—is higher in the Mixed-Conjunction block. Therefore, it is plausible that the slowing of RTs is not due to modulation of proactive control per se, but rather reflects increased general attentional engagement, which could also account for the improved lane-keeping performance.

A detailed response to Comments 2, 3, 4 and 6 has been provided together in response to Comment 6.

4. In the introduction, the authors cite literature on both proactive and reactive suppression of distractors. However, their paradigm does not allow for a direct manipulation of these two mechanisms. It is entirely possible that, due to its similarity with the target, the distractor in the Mixed-Conjunction block captures attention, and that the observed cost reflects a reactive suppression process that reallocates attention to the target.

A detailed response to Comments 2, 3, 4 and 6 has been provided together in response to Comment 6.

5. To support a claim of proactive control being engaged to suppress distractors, physiological evidence (e.g., EEG or eye-tracking) would be necessary—see, for example, the work by Nick Gaspelin.

We acknowledge that current data do not provide enough evidence to support strong claims about the engagement of proactive control processes (but see also response to comment 6). Therefore, we agree that additional neurophysiological measures (e.g., EEG) would be necessary to directly address this issue. In the revised manuscript we have explicitly acknowledged this limitation in the following paragraph:

LINES 549-558: *“**While firm conclusions about the precise mechanisms underlying our findings (e.g., the involvement of negative attentional templates) cannot be drawn from this speculation, we consider it is more likely that a proactive mechanism, rather than a reactive one, accounts for our data. This interpretation is supported by the analysis on braking RTs in TP-DA trials, where the absence of distractors rules out the involvement of reactive distractor suppression mechanisms. Furthermore, given that TP-DA trials were physically identical across blocks, any difference in RTs can be attributed to varying levels of distractor features and associated expectations. To address this open question, neural activity measures (e.g., EEG) would be beneficial, particularly through time-frequency analysis focusing on theta oscillations over frontoparietal regions, theta phase synchronization in prefrontal regions and posterior alpha power [39].”*

6. Behaviorally, could the authors show that, as distractors repeat, reaction times to detect the target become progressively faster? This would support the idea of learned suppression (Gaspelin & Luck), which is believed to rely on proactive control mechanisms.

As the Reviewer proposed in comments 2 and 3, we agree that varying levels of vigilance and/or cognitive demand across blocks may have affected accuracy and response speed. We also agree with the Reviewer that the Mixed-Conjunction block, being more cognitively demanding, could have taxed sustained attention (or general attentional engagement) more heavily.

While we cannot completely rule out this possibility, as our setup did not include a direct sustained attention measure, we believe it cannot fully explain slower RTs, for the following reasons:

1. While increased vigilance typically speeds up RTs, especially at the beginning of a block, if RTs modulation were fully explained by fluctuations in vigilance, we would expect RTs to progressively slow down as the block progresses, an effect known as vigilance decrement (see McCarley & Yamani, 2021). Additionally, if vigilance levels vary as a function of cognitive demand, we would expect the rate of vigilance decrement to differ across Pure, Feature, and Conjunction Search blocks, as well as between TP-DA and TP-DP trials, because being physically different, they would likely produce distinct responses. To test this prediction, we ran two independent mixed-effects linear regression models, one on TP-DA trials and one on TP-DP trials. We included polynomial contrasts up to the cubic term for Trial sequence and Block Type as fixed effects, with a random intercept for subjects. The main results of this analysis are reported herein, while the detailed analysis is presented in Supporting Information.

Results for TP-DA trials indicated a significant main effect of the cubic polynomial for Trial sequence (right graph; F_(3, 2073.1)_ = 4.27, *p* = .005) as well as for Block Type (F_(1, 2073.0)_ = 78.9, *p* < .001), while their interaction did not reach statistical significance. Specifically, the analysis of the orthogonal polynomial contrasts revealed a significant cubic component (β = -0.58, SE = 0.29, t_(2073)_ = -2.00, *p* = .045) while neither the linear nor the quadratic components did reach significance (*p* > .05). Independent analyses on each block revealed a significant cubic component for both the Mixed-Conjunction (β = -0.33, SE = 0.17, t_(669)_ = -1.98, *p* = .048) and the Mixed-Feature (β = -0.28, SE = 0.14, t_(677)_ = -2.03, *p* = .043), and a significant quadratic trend for the Pure Block (β = 0.54, SE = 0.15, t_(681)_ = 3.71, *p* < .001).

The Mixed-Conjunction Block elicited the slowest RTs overall, but RTs tended to decrease as the block progressed. A similar decrease was observed for the Mixed-Feature Block. In the Pure Block, RTs initially decreased but returned to the initial level at the end of the block. One possible explanation is that in the Mixed-Blocks participants began to anticipate TP-DA trials toward the end, thereby mitigating the negative impact of distractor expectations when such expectations were not met. This analysis confirms a progressive and nonlinear decrease in RTs across trials in both the Mixed-Conjunction and Mixed-Feature blocks, consistent with the hypothesis of proactive anticipation of TP-DA trials by the end of the blocks. In contrast, the “U-shaped” pattern observed in the Pure block, which included only DA trials, may be explained by reduced vigilance toward the end of the block, induced by its monotonic nature.

It might be argued that RTs already differed at trial 1. However, it should be noted that each block was preceded by a one‑minute training period, allowing participants to preview the task and develop a distractor expectation set. This familiarization phase likely contributed to establishing a baseline before the actual experimental session began.

Results for TPDP trials indicated a significant main effect of Block Type (F_(1, 2776.0)_ = 167.26, *p* < .001) and a significant interaction between Block Type the quadratic trend for Trial sequence (F_(3, 2776.0)_ = 2.82, *p* = .037). Specifically, the analysis of the orthogonal polynomial contrasts revealed that the Mixed-Feature Block entails a significant quadratic component as compared to the Mixed-Conjunction one (β = 0.65, SE = 0.32, t_(2778)_ = 2.00, *p* = .046). Independent analyses on each block revealed a significant quadratic component for both the Mixed-Feature (β = 0.41, SE = 0.15, t_(1382)_ = 2.75, *p* = .006) and non-significant trends for the Mixed-Conjunction one (*p* > .05).

While the difference between the two blocks may be attributable to differences in cognitive demands, we hypothesize that this result is primarily due to our task design. Specifically, we employed an overt visual search with long exposure to the stimuli, which is optimal for observing variability in response times. However, even though participants were instructed to break to target stimuli as promptly as possible, the long exposure time might have allowed enough time to do this task while reactively, rather than proactively, suppressing stimuli, which would explain the lack of a learned suppression. To keep the main text focused and concise, we decided to report these additional analyses in Supporting Information, where they are fully available for interested readers. However, we remain open to the Editor’s or reviewers’ suggestions, should they prefer these analyses to be incorporated into the main text.

2. Additionally, it should be noted that RTs for braking have been analyzed on TP-DA trials across blocks, that is, on physically identical trials free of distractors. The cognitive demands of these trials are inherently different from those of TP-DP trials. We believe that, regardless of the exact mechanism at play, the key factor underlying the observed effect is the expectation that a distractor will appear on trial n+1. Accordingly, these differences cannot be explained by varying levels of cognitive demand or by reactive mechanisms. Rather, if anything, it is the “expectation” of higher cognitive demand that may disrupt performance when those are not met, an effect we hypothesize to be proactive, although additional methods (e.g., EEG, see also response to comment 5) would be required to directly address this question.

In the revised manuscript we have toned down the emphasis on proactive mechanisms as we acknowledge these remain hypothetical as current data do not provide sufficient evidence to support this claim. Specifically, we’ve addressed these points in the following paragraph:

LINES 559-584*: “It could be argued that our findings might alternatively be explained by differences in task demand and cognitive load, stemming from the adoption of different strategies to exploit the search task. Specifically, whereas Feature Search primarily relies on bottom-up perceptual distinctiveness between target and distractors [27,69], Conjunction Search – where target selection and/or distractor suppression are largely, though not exclusively, driven by top-down processing [70,71], engages more controlled mechanisms, thus making this type of search more cognitively demanding.*

*While this represents a plausible alternative framework, we believe it can be dismissed for three reasons: (i) if the modulation of our observed RTs were fully attributable to fluctuations in vigilance, we would expect RTs to progressively slow down over the course of a block, an effect referred to as vigilance decrement [72]. Furthermore, if vigilance levels are scaled as a function of cognitive demand, the rate of vigilance decrement should vary across Pure, Feature, and Conjunction Search blocks, as well as between TP-DA and TP-DP trials, because being physically different, they would likely elicit distinct responses. However, our data do not support this prediction (see Supporting Information); (ii) although our Braking RTs results suggest that the observed effect is modulated by the visual search complexity imposed by distractors features, it should also be noted that this analysis was conducted on TP-DA trials, where no distractors were present on-screen. Therefore, since the comparison across blocks was based on physically identical trials, the observed differences are unlikely to reflect varying task demands or reactive distractor suppression mechanisms, but rather differences in attentional sets linked to distractor expectations; accordingly, RTs differences might stem from varying levels of expectation about the trial n being more or less demanding, rather than from a pure effect of task demand; accordingly, if anything, it is the “expectations” of higher cognitive demand to impair performance when those are not met, an effect we hypothesize to be proactive, although additional methods (e.g., EEG) would be required to directly address this question; (iii) if task demand had truly affected driving performance, a negative impact should have been observed in Lane-Keeping for Mixed (vs. Pure) Blocks. Instead, we found that merely expecting distractors improved drivers’ ability to maintain optimal vehicle positioning along the route.”*

Overall, I would recommend that the authors revise their manuscript not around the concept of proactive control per se, but rather more broadly around distractor suppression and its interference with driving performance. As currently presented, the evidence for proactive control is not sufficiently convincing to warrant such strong claims.

**Reviewer #2:**

The subject of this study is quite engaging, and I have several recommendations to enhance its quality:

1. The simulation employs Carnet software. It would be advantageous for the authors to incorporate more details regarding the road environment, weather conditions, and traffic scenarios.

A detailed description of the CarnetSoft software is presented in the following paragraph:

LINES 251-257: “*The virtual environment consisted of an extra-urban two-lane road within a daytime rural landscape, with a mostly sunny sky and a few scattered clouds. Drivers experienced a right-hand traffic (RHT) first-person racing perspective. The road environment included task-irrelevant lateral road signs and other vehicles travelling in the same direction. Audio-visual stimulation consistent with the environment was provided throughout the simulation to render the driving experience engaging and realistic. The road circuit was a one-way semi-circular loop, almost entirely rectilinear, with sharper bends at its extremities, allowing the drivers to re-enter the route (see Fig. 1b). ”*

2. I recommend verifying the standards used for the testing process. For example, the simulation speeds are set at 50 and 80 km/h. What criteria were considered in selecting these specific speeds?

Driving-related parameters were chosen in accordance with previous studies employing similar driving simulations and experimental paradigms (see Karthaus et al., 2018; 2020 & Facchin et al., 2023). This information is now reported in:

LINES 242-243: *“Importantly, in line with previous studies using similar driving paradigms (see Karthaus et al., 2018; 2020 & Facchin et al., 2023), we set precise constraints on specific driving-related parameters to comply with the need for experimental control.”*

3. In my view, a three-minute training session for new users is inadequate, as the simulator's operation significantly differs from that of a real vehicle. Please offer your justifications for this training duration.

We understand the Reviewer’s concern. The training was designed to familiarize participants with the driving simulator setup, particularly the steering wheel sensitivity and the brake pedals and did not involve any visual search task. The choice of three-minute training was based on pilot testing conducted prior to the actual data collection. Furthermore, in Facchin et al. (2023), a five-minute training session preceded the experimental session. In our study, each block was also preceded by an additional one-minute training period to preview the task. If participants had required additional familiarization with the setup, the training could have been repeated; however, this was never necessary. This information are reported in:

LINES 326-330: *“Similarly to previous studies (see Facchin et al., 2023), participants completed a three-minute pre-experiment training session. The training session was aimed at familiarizing participants with the driving simulator setup and, specifically, at allowing them to adapt their movements to the sensitivity of the steering wheel and the pedal board. No visual search task was included in the training. Moreover, the training could be repeated if additional familiarization was needed, but this was never required.“*
