# Supplementary material for: Prepared for the expected but unready for the unexpected: Unmet distractor expectations slow braking responsiveness but improve lane-keeping precision in a virtual driving simulation
Source: PLoS One. 2025 Dec 26;20(12):e0338124. doi: 10.1371/journal.pone.0338124 (PMC12742790; doi:10.1371/journal.pone.0338124)
Supplement: Attachment — Submitted filename: ResponsesToEditor_PLOS.docx [file pone.0338124.s006.docx]

**Journal Requirements:**

1. If the reviewer comments include a recommendation to cite specific previously published works, please review and evaluate these publications to determine whether they are relevant and should be cited. There is no requirement to cite these works unless the editor has indicated otherwise.
2. Please review your reference list to ensure that it is complete and correct. If you have cited papers that have been retracted, please include the rationale for doing so in the manuscript text, or remove these references and replace them with relevant current references. Any changes to the reference list should be mentioned in the rebuttal letter that accompanies your revised manuscript. If you need to cite a retracted article, indicate the article’s retracted status in the References list and also include a citation and full reference for the retraction notice.

**Additional Editor Comments:**

**Background**

This is a kind of dual-task study conducted in the context of simulated driving, where a primary task required participants to steer a car with a Logitech wheel and pedal simulator on a curved two-lane road, and a secondary task required them to watch for a target (a standard red stop road sign), and to press the brake pedal as soon as it appeared. Three conditions were tested, in the first, only the normal target (stop road sign) appeared, and participants where to brake whenever it was shown, in the second, the real stop sign was mixed with colour-altered signs that looked similar but were not true stop signs, and in a third condition, the real stop sign was mixed with more complex variations, where the colours and layout were changed, making them visually confusing but still non-targets. In every case, participants were instructed to brake only for the standard red stop sign and to ignore all others. The results showed that when these altered signs appeared frequently in the inter-mixed conditions, participants became slower to brake even when the true stop sign appeared, but at the same time they kept the car more precisely within the lane. This indicates that expecting possible distraction delays the braking response yet improves the readiness for steering control.

**Evaluation:**

This study is clearly a laboratory-based experiment, and as such it represents a controlled but necessarily simplified model of real driving. The simulated setting does not reproduce all aspects of driving a real vehicle, which limits the degree of ecological realism. At the same time, it is evident that modelling genuine driving is inherently difficult, as one must decide which features of the task are critical to retain while keeping the situation experimentally manageable. In this respect, the present study achieves a reasonable balance. The simulator provides a physically embodied steering and braking task, which captures the continuous control demands of real driving while still allowing for precise experimental manipulation. The sample size (N = 24) is modest but acceptable for this kind of within-subject design, where each participant contributes a large number of trials. The manipulation is well structured and internally consistent: one condition involves responding only to the true stop sign, while the mixed conditions introduce non-target events that vary in visual similarity to the target. The essential logic of the design is sound, but see my detailed comments.

**(1) some basics: abstract and title**

Abstract: The abstract is nearly 300 words long, too wordy, and overly narrative. It contains several passages that are not typical for a scientific abstract and that obscure rather than clarify the main message. I recommend shortening it substantially, and restructuring it around four clear elements: what is aimed, what is done (rationale, design, factors, and measures), what is found, and what this means conceptually (the take-home message).

In the revised paper, we have substantially restructured the abstract, reducing it from 244 to 199 words. Additionally, we have made it more schematic and less narrative. While organizing it around the four elements suggested by the Editor, we have still retained a brief theoretical background, as we believe it helps the reader to quickly position the paper within the relevant literature.

*Driving requires attentional control mechanisms to enhance the detection of driving-relevant objects and to mitigate interference from distractors. While distractor suppression can be proactively engaged when distracting events are expected, this mechanism also comes along with both costs and benefits, affecting responsiveness and accuracy even in their temporary absence. We explored the effect of distractor expectations on driving performance. Through an immersive driving simulator, participants (N = 24) performed an adapted version of the Distractor Context Manipulation (DCM) paradigm. They navigated a circuit and promptly pressed the brake pedal whenever a road sign-like target appeared, under three different conditions: a Pure Block without distractors, and two Mixed Blocks, featuring frequent irrelevant distractors (67% of trials) differing in perceptual complexity (Feature Search vs. Conjunction Search). We measured braking RTs and lane-keeping precision. Results showed that Mixed Blocks delayed braking RTs, even when distractors were temporarily absent, with the magnitude of this delay scaling with the perceptual similarity between target and distractors. Conversely, the same mechanism improved lane-keeping precision when neither targets nor distractors were present. Our findings suggest that distractor expectations modulate driving performance, entailing a trade-off between responsiveness and precision that depends on the characteristics of the distractor context.*

Should the Editor deem it more appropriate, we would be happy to explicitly restructure the abstract in different and delineated sections (e.g., Background, Aim, Methods, Results, and Conclusions).

Title: The current title (Optimizing driving performance: the impact of distractor expectations on braking and lane-keeping) is somewhat misleading. The study does not involve optimisation of real driving, nor does it test distractor expectations in the applied sense implied by the title. Instead, it examines a dual-task situation combining a continuous primary task (background task: lane keeping) with a secondary prospective-event task (braking to stop road signals). I therefore recommend rewording the title to reflect this conceptual focus. Something short and memorable that conveys the “just do it when you get a chance” logic of the paradigm would better capture the actual content and originality of the study.

We have revised the paper’s title to better emphasize the task’s duality and tone down any reference to driving optimization. Also, we have rephrased it to make it more memorable by adding a catchy introductory phrase, which lightly lengthens it. While we believe this version is worth retaining, we are happy to adopt only the second part of the title should the Editor consider it more appropriate:

*“Prepared for the expected but unready for the unexpected: unmet distractor expectations slow braking responsiveness but improve lane-keeping precision in a virtual driving simulation”*

**(2) Ecological validity**

This is clearly a laboratory experiment, and it does not represent real driving in any strict sense. It uses artificial visual events that would never occur in real traffic, and it isolates them from the contextual cues that normally accompany such events. In reality, a stop sign appears at an intersection, surrounded by situational markers that jointly define its meaning. In the present simulation, by contrast, stop-sign stimuli appear at arbitrary positions along a straight segment, outside any traffic context. The manipulation therefore represents a situation that is not merely decontextualised but almost contra-situated relative to genuine driving. From the standpoint of experimental logic, this is legitimate, yet it shows that the factorial structure of the study is conceptually removed from the ecological conditions it nominally models. On the other hand, within the limits of a laboratory, the study is remarkably well implemented and technically accomplished. The use of a Logitech G29 wheel and pedal system provides continuous sensorimotor feedback, and the visual immersion is sufficiently rich to induce a sense of genuine control. In this respect, the study reaches the upper bound of what can be achieved within a behavioural-laboratory setting. It captures the feeling of driving through steering, braking, and lane keeping, even if the external events remain abstract.

Conceptually, the design follows the logic of a continuous dual-task paradigm, where a primary task (lane maintenance) is performed as background while a secondary task (braking to infrequent events) occurs intermittently. In this way, the structure parallels prospective-memory designs in which participants respond to an occasional cue “when they get a chance” while engaged in another ongoing activity (e.g., Janczyk, Durst, & Ulrich, 2017; Miller & Durst, 2014). I suggest that this relation be made explicit in the manuscript, since one critical aspect of such paradigms is the temporal spacing of events, which determines the balance between sustained readiness and periodic re-engagement. Finally, constructing a study that genuinely represents real driving is not only a technical issue but also a deeper philosophical problem. Brunswik (1955), in his notion of representative design, already noted that every experimental model requires a decision about which elements of the natural situation to preserve and which to emphasise at the expense of others that are simplified or muted. This choice defines what kind of knowledge an experiment can produce. To put it simply, different designs resemble different types of maps: one may depict political borders in colour, another geological layers through shading, another may adopt a topological rather than a topographical representation. Each offers a partial but purposeful view of the same reality. I would suggest elaborating on this point in the discussion, as such a reflection would make the paper intellectually stronger and conceptually deeper.

Brunswik, E. (1955). Representative design and probabilistic theory in a functional psychology. Psychological Review, 62(3), 193-217. doi:10.1037/h0047470

Janczyk, M., Durst, M., & Ulrich, R. (2017). Action selection by temporally distal goal-states. Psychonomic Bulletin & Review, 24(2), 467-473. doi:10.3758/s13423-016-1096-4

Miller, J., & Durst, M. (2014). "Just do it when you get a chance": the effects of a background task on primary task performance. Attention Perception & Psychophysics, 76(8), 2560-2574. doi:10.3758/s13414-014-0730-3

The insight provided is correct: under some circumstances, our task may be configured as a dual-task. Specifically, this occurs on TP-DA and TP-DP trials, where participants perform a primary task (lane keeping) in the background while a secondary task (braking to targets) is performed. The Editor suggested that, accordingly, our task design parallels the structure of prospective-memory ones in which participants respond to an occasional cue “when they get a chance” while engaged in another ongoing activity, and he cited, among others, the Miller & Durst (2014) paper. In their work, they exposed participants to a “prioritized-processing paradigm”, which comprises a high-priority primary task that was performed only when an action was required, and a low-priority background task performed only when no action was needed for the high-priority primary task. The authors demonstrated that high-priority primary task performance in the multitasking condition was compromised compared to when the task was performed in isolation. Given such an assumption, the following prediction logically follows for our task: if multitasking conditions make performance worse on the primary background task, then the lane-keeping enhancement observed in TA-DA trials of the Mixed Blocks (i.e., when only lane maintenance had to be performed), as compared to the Pure one, should disappear in TP-DA and TP-DP trials (i.e., when both lane maintenance and braking to targets had to be performed). To formally test this prediction, we replicated the main analyses on lane-keeping TA-DA trials, this time on both TP-DA and TP-DP trials. Crucially, results showed a significant interaction between Block Type and Road Curvature Variability (F_(2, 4887)_ = 6.25, *p* = .002). As in the TA-DA trials analysis, Road Curvature Variability negatively impacted Lane-Keeping across all blocks. Moreover, mirroring those results, this effect was mitigated in both the Mixed-Feature (b_Mixed-Feature Block_ = 0.732, SE_Mixed-Feature Block_ = 0.004) (t_(4887)_ = -3.359, *p* = .024) and the Mixed-Conjunction (b_Mixed-Conjunction Block_ = 0.732, SE_Mixed-Conjunction Block_ = 0.004) (t_(4887)_ = -3.246, *p* = .024) Blocks as compared to the Pure Block (b_Pure Block_ = 0.757, SE_Pure Block_ = 0.007). Ultimately, the Mixed-Feature vs. Mixed-Conjunction Blocks contrast was not statistically significant (t_(4889)_ = 0.069, *p* = .945).

Therefore, these results indicate that Mixed Blocks, regardless of the type of distractor expected, mitigate the negative impact of driving demands on lane maintenance, even when a secondary, intermittent task must be performed. Should the Editor consider it relevant and appropriate, we would be happy to add this additional analysis to the Supporting Information material.

As the lane maintenance performance was consistent across both TP and TA trials, it is unlikely that our results are due to multitasking interference or to the temporal spacing of events, which, as the Editor suggested, may determine the balance between sustained readiness and periodic re-engagement. Additionally, it is worth noting that in our task, braking never involved a complete stop, implying that lane maintenance had to be maintained from the beginning to the very end of the task, even when braking was required. Ultimately, while multitasking interference is often linked to response compatibility, both our tasks, namely lane maintenance and braking, require different effectors: while lane maintenance involves hand responses, braking is instead implemented through foot responses. Moreover, while braking was implemented unilaterally (i.e., typically through the right foot), lane maintenance was implemented bilaterally (i.e., through both hands; the instruction was explicitly provided to participants at the beginning of the experiment), thus mitigating possible interference arising from ipsilateral vs. contralateral motor schemas.

We fully agree that ecological validity is a critical consideration for our experiment. This issue is well recognized in the vision science literature (see, for example, Lappi, 2018, for a review). Capturing aspects of real-world driving can indeed be pursued through a range of approaches, from highly controlled laboratory experiments to field experiments. As already acknowledged in our previous response, the necessary trade-off between external validity and experimental control limits the extent to which our findings can be generalized to real-world driving situations, characterized by more dynamic, entropic, and unpredictable events. We also recognize that this inevitable balance implies the need to clearly state to what specific aspect of driving our experimental design maps into, and why it was necessary to sacrifice certain key aspects of ecological realism that inherently define the driving experience. Unlike many laboratory tasks, typically defined by simplified and abstract stimuli to isolate a single cognitive function, driving is intrinsically a complex, integrative activity: it requires the orchestration of distinct cognitive and sensorimotor functions, including (but not limited to) foveal and peripheral vision, spatial reasoning, memory, motor planning, task switching, monitoring, and prediction. These functions jointly enable the driver to efficiently detect, elaborate, and respond to unexpected and dynamic events. However, isolating one of these functions to understand its specific contribution to the overall driving process necessarily requires retaining just a few elements of the driving complexity at the expense of others, which could potentially confound the results.

Our primary objective was to investigate the impact of distractor expectations on driving performance by adopting a well-validated, standard laboratory task and adding (some) features of real-world driving, focusing specifically on two key aspects: a general driving performance indicator, the ability to maintain the vehicle within the lane, and the ability to promptly brake in response to abrupt, relevant events. While the first is fundamental across a wide range of driving situations, even in the absence of other driving-relevant objects (e.g., other cars or pedestrians), and can therefore be replicated with a high degree of ecological validity also in well-controlled experimental contexts, the latter typically occurs only in specific driving contexts (e.g., a child suddenly crossing the street without looking around before, a cyclist running a red light, a tree branch falling onto the road during heavy rain and strong wind). Moreover, the likelihood of such events varies depending on the driving context: for example, some may occur along straight roads, whereas others are more common at intersections. In the real world, each driving context carries its own set of expectations along with informative cues (e.g., warning signs) and individual drivers’ experiences. Controlling for these factors is therefore essential to draw reliable conclusions about the general impact of distractor expectations on driving performance. For this reason, employing a generic and neutral driving environment, along with neutral stimuli for which participants must learn the associated response, was, in our view, the most methodologically sound approach. Accordingly, we believe that our study maps into very general and fundamental aspects of driving, which are likely to be shared across a variety of situations that may occur at the wheel.

At the same time, we acknowledge that our findings cannot be generalized to more specific and ecologically valid contexts, where additional factors may differentially modulate these mechanisms. Nevertheless, we consider that understanding these mechanisms at a broader level is important for addressing more specific driving situations and for informing both theoretical and applied research.

In the revised manuscript, we addressed these issues in the following paragraphs (see lines 613-644):

*“Despite its contributions, our study still presents a relevant limitation that must be considered. In particular, while this study represents an important step toward scenarios that more closely approximate the perceptual and motor demands of real-world driving, it still involves a relatively high level of experimental control and artificial driving demands, which constrain the direct generalizability of our findings to real-world driving contexts. Vision science has long recognized the methodological challenge of modelling real-world driving conditions (see, for example, [16], for a review). Capturing its fundamental aspects can indeed be achieved through a continuum of approaches, from highly controlled laboratory experiments to naturalistic field studies, each reflecting a different balance between external validity and experimental control. It may thus be argued that our task does not reflect genuine driving performance, and that the driving component merely serves as a context for a standard lab-based experiment on the cognitive processes underlying distractor’s expectations. However, our setup utilizes a markedly different approach from standard laboratory tasks [43], that is a driving simulation featuring a dynamic and context-rich visual environment. Additionally, the response measures are inherently naturalistic driving actions, whose motor demand differs significantly from button-press responses common in laboratory settings. Specifically, the first critical measure, timely braking in response to abrupt, context-dependent events, reflects a natural, safety-relevant action which typically occurs only in specific scenarios (e.g., a child crossing the street, a cyclist running a red light, or a tree branch falling onto the road during heavy rain and strong wind), whereas the second, lane-keeping, is typically absent in standard experimental design. Even more importantly, lane-keeping was recorded and analyzed in the absence of any attentional and visual demand unrelated to driving. Ultimately, the use of driving simulations and motor responses that reflect real-world driving tasks has been recognized as a scientific standard, as evidenced by numerous studies investigating attentional processes at the wheel [4,6,9,52–54,73]. While lane-keeping can be reliably assessed even in highly controlled settings, context-specific events vary in likelihood, and the way drivers act upon them is shaped by environmental cues and prior experience. Given this variability, and to ensure methodological rigor, we employed a generic driving environment with neutral stimuli that drivers had to learn how to respond. This approach allowed us to map onto general and fundamental aspects of driving performance that are likely generalizable across a wide range of real-world scenarios.*

*Collectively, these methodological aspects demonstrate that participants were fully engaged in a genuine driving-related task, with behavioral responses directly reflecting cognitive and motor demands inherent to real world-driving.”*

**(3) Theoretical mechanisms**

The study comprises three conditions. In the first (pure) condition, participants performed the primary task and were instructed to brake whenever the secondary, prospective-event signal (the standard red stop sign) appeared. This condition already involves a temporal expectancy mechanism: after each occurrence, readiness rises, is actively maintained for a short period, and then decays gradually across subsequent trials. In the two mixed conditions, additional events (decoy stop-road signs) were interspersed. Although these decoy signals did not require overt braking, they nevertheless required stimulus evaluation and thus engaged the same detection process. In this way, they function conceptually as decoy prospective-event signals. R1 already noted that adding such decoy events among the normal targets increases the rate at which participants must pause, evaluate, and process incoming stimuli, and this is in fact the critical variable at work in the present design. With shorter event-to-event distances, the expectancy window is repeatedly reactivated, keeping participants in a state of continuous readiness for potential braking. This pattern means that the observed response slowing in the mixed conditions has nothing to do with feature-conjunction search or feature-integration mechanisms and does not reflect interference from distractor features. It rather reflects the sustained activation of the braking schema due to the higher frequency of prospective events. In other words, the shorter event-to-event distance keeps participants effectively in a be-ready-for-braking mode. I suggest that the authors reconsider the theoretical rationale of the study not in terms of feature similarity or conjunction complexity, but in terms of the temporal structure of expectancy, effort mobilisation, and the maintenance of active readiness (see Schumann et al., 2022, chap. 4.5, for a theoretical analysis). Elaborating on this mechanism in a revision would substantially deepen the theoretical coherence of the manuscript.

Altmann, E. M. (2002). Memory for goals: an activation-based model. Cognitive Science, 26(1), 39-83. doi:10.1207/s15516709cog2601_2

Altmann, E. M. (2002). Functional decay of memory for tasks. Psychol Res, 66(4), 287-297. doi:10.1007/s00426-002-0102-9

Schumann, F. et al. (2022). Restoration of attention by rest in a multitasking world: Theory, methodology, and empirical evidence. Frontiers in Psychology, 13, 867978. doi:10.3389/fpsyg.2022.867978

We thank the Editor for this comment, which allows us to further clarify this point. First, it should be noted that our primary aim was not to validate a new paradigm to demonstrate that it captures control mechanisms for distractor expectations, a goal that has already been successfully addressed by others (see Marini et al., 2013, 2016; Ravagli et al., 2018; Petilli et al., 2020). Rather, we aimed to show that the same effects reported in previous studies persist under more naturalistic, yet experimentally controlled, conditions, specifically, in a driving simulation. However, we acknowledge that all the effects mentioned in the suggested literature are relevant and could, in principle, contribute to the observed results. However, we believe we can confidently rule them out for the following reason: (1) it is true that, theoretically, adding decoy events increases the rate at which stimuli must be detected, evaluated, and eventually acted upon. It is also true that our Mixed Blocks include three times the number of trials as compared to the Pure Block. However, it should also be noted that Mixed Blocks were divided into three sub-blocks, each made of 60 trials, to match the length of the Pure Block. Consequently, the rate of event onset was identical for all three critical conditions, allowing us to rule out any potential effect due to the temporal spacing between events. (2) On top of that, the activation of the braking schema was identical in all conditions: in both the Pure and the Mixed Blocks, the braking response was required on 50% of the trials. Therefore, we can exclude the possibility that the slowing in RTs arises from a greater, sustained activation of the braking schema. (3) All our results on braking RTs are based exclusively on TP-DA trials, i.e. trials in which only the same target was displayed. Therefore, differences across conditions are based on physically identical trials, which differ only in their block context, specifically whether distractors were expected to appear more frequently and, if so, how distinguishable they were from the target. This manipulation, indeed, directly affects search efficiency and the relative contribution of feature vs. conjunction-based processes. (4) We also acknowledge the Editor's point that the slowing observed in the mixed conditions could, in principle, stem from a higher frequency of events requiring stimulus evaluation, which may increase temporal expectancy and sustained activation of the braking schema. Although this interpretation is entirely reasonable, it does not fully account for the behavioural pattern consistently reported (and replicated in the present study) in other studies using the very same paradigm adopted here. Indeed, analogous effects have been observed even in the absence of differences in the frequency of events requiring evaluation. In fact, this behavioural pattern has already been demonstrated across multiple experiments involving various tasks that validated the very same procedure implemented here, including studies in which the frequency of events requiring stimulus evaluation was kept identical to ours, and others in which it was constant across conditions. Across all such cases, the same pattern reliably emerged. For instance, in Experiment 4 of Petilli et al. (2020, Cognition), all trials required a speeded response, thereby eliminating differences in event-to-event intervals and evaluation demands. Yet, the same proactive slowing was observed when distractors were expected but not presented, demonstrating that top-down distractor anticipation elicits sustained cognitive control costs in visual search (along with a corresponding speed–accuracy trade-off; for a comparison of the effects, see also Experiments 1, 2, and 3 of Petilli et al., 2020). This proactive slowing has also been systematically replicated in paradigms that did not introduced additional events requiring evaluation in the Mixed (vs. Pure) block, including crossmodal visuo-tactile designs across different sensory modalities and contextual manipulations (Experiments 1–5 and 7 in Marini et al., 2013, Journal of Experimental Psychology: General) and flanker tasks (Marini et al., 2016, Journal of Neuroscience; see also Experiment 6 in Marini et al., 2013). This suggests that the effect is not contingent on increased response-related activation or more frequent stimulus evaluation. Instead, it reflects cognitive mechanisms associated with proactive control processes triggered by increased distractor expectation in Mixed blocks. Building upon this robust body of evidence, which validated the procedure and firmly established that the slowing effect in the Pure block results from proactive control driven by distractor expectations, rather than event-pacing differences, our study extends these mechanisms to a more ecologically valid yet experimentally controlled context: a driving simulation. We showed that proactive costs associated with distractor expectations persist and manifest as a characteristic pattern of driving behavior when embedded in realistic scenarios. Thus, although the mechanism proposed by the Editor is certainly plausible, the interpretation we advance is more strongly supported by prior work specifically designed to examine and isolate the cognitive mechanisms involved in this paradigm, and therefore provides a more robust explanation of the observed behavioral pattern.

We thank the Editor for this insightful comment, and we now clarify this aspect explicitly in the revised manuscript (see lines 582-595):

“*Notably, the slowing observed in the Mixed Blocks could alternatively reflect the higher frequency of prospective-event signals requiring stimulus evaluation, which may help maintain a sustained state of braking readiness throughout the block. In principle, such an increased rate could prolong the activation of this readiness state and, potentially, delay responses to actual braking signals. However, this account does not fully capture the pattern of findings reported across studies employing the same paradigm used here. Indeed, proactive slowing has been consistently observed across a wide range of laboratory tasks, even when the frequency of events requiring stimulus evaluation is matched across blocks (e.g., crossmodal visuo-tactile designs, [40]; flanker tasks, Marini et al. [40,41]; visual search, Experiment 4 in Petilli et al. [43]). These converging findings demonstrate that the observed cost cannot be attributed to a sustained braking schema driven by higher event pacing. Instead, it more likely reflects proactive control processes triggered by an increased expectation of distractors. In line with this interpretation, the present study shows that proactive-control costs associated to distractor expectations persist and translate into a characteristic pattern of driving behaviour when embedded within realistic driving simulations.*”
